# Electrocardiographic Abnormalities in Patients With Leptospirosis: A Clinical Observational Study

**DOI:** 10.7759/cureus.98356

**Published:** 2025-12-03

**Authors:** Kavin Pranav B, Sahasyaa Adalarasan, Yogesh S, Prithvi Nikesh, Hariharan C

**Affiliations:** 1 Institute of Internal Medicine, Madras Medical College, Chennai, IND; 2 Health Profession Education, Sri Balaji Vidyapeeth University, Puducherry, IND; 3 Internal Medicine, Madras Medical College and Rajiv Gandhi Government General Hospital, Chennai, IND

**Keywords:** ecg, ecg changes, leptospira interrogans, leptospirosis, weil's disease

## Abstract

Background: Leptospirosis is a globally prevalent zoonotic disease with diverse clinical manifestations, including potentially significant cardiovascular involvement. Electrocardiography (ECG) represents a convenient, cost-effective tool for detecting early myocardial and conduction abnormalities in affected patients.

Methods: In this observational study, 108 serologically confirmed leptospirosis patients underwent standard 12-lead ECG recording upon admission. Demographic characteristics, serum sodium, and potassium levels were documented. Patients with pre-existing cardiac disease were excluded. ECG abnormalities were categorized, and their distribution was analyzed using descriptive statistics and correlation tests where appropriate.

Results: The mean age of the cohort was 34 ± 4.76 years with equal sex distribution. Fourteen patients (13%) died, and 43 (40%) required ICU admission. Hyponatremia (mean = 132 mEq/L) and hypokalemia (mean = 3.1 mEq/L) were common. ECG abnormalities were observed in a majority of patients, with non-specific ST-T changes and myocarditis-like patterns being most frequent, followed by left ventricular hypertrophy-like changes with sinus tachycardia, atrial fibrillation, and primary atrioventricular (AV) block. QTc prolongation was uncommon. Myocarditis-pattern ECG changes showed a significant association with disease severity.

Conclusion: ECG abnormalities are common in leptospirosis and may serve as early markers of myocardial involvement and disease severity. Routine ECG evaluation, particularly in severe disease or electrolyte derangement, can assist in timely management. Further studies integrating echocardiography and long-term outcomes are needed to delineate the prognostic value of these findings.

## Introduction

Leptospirosis is a widespread zoonotic infection caused by pathogenic spirochetes of the genus *Leptospira*, species *Leptospira interrogans*. These motile, corkscrew-shaped bacilli are capable of penetrating abraded skin and mucous membranes, entering the bloodstream, and disseminating rapidly [1]. Human infection typically occurs through occupational or environmental exposure to water or soil contaminated with the urine of infected rodents and other reservoir animals [2]. Consequently, outbreaks are common in agricultural settings such as rice fields and in regions experiencing heavy rainfall or flooding, conditions that promote prolonged organism survival and increased human contact [2].

Clinically, leptospirosis classically exhibits a biphasic course. The initial septicemic phase is characterized by high-grade fever, myalgia, conjunctival suffusion, headache, and nonspecific constitutional symptoms. This is followed by the immune phase, in which patients may develop meningitis, hepatic dysfunction, renal impairment, or possibly even pulmonary involvement [3]. A minority of individuals progress to Weil’s disease, the fulminant and severe form of leptospirosis marked by jaundice, acute kidney injury, hemorrhagic tendencies, and multiorgan dysfunction. Mortality in Weil’s disease remains high, particularly in patients with cardiac or pulmonary complications [3].

The pathophysiology underlying severe leptospirosis is multifactorial, with the likely cause being the disruption of renal tubular ion transport. Specifically, interference with Na⁺-K⁺-Cl⁻ cotransporter function in the nephron has been proposed, leading to impaired electrolyte handling [4]. As a result, patients frequently develop disturbances such as hypokalemia, hyponatremia, and metabolic derangements, each of which can predispose to significant electrical instability within the myocardium.

Cardiac involvement in leptospirosis is diverse and often underrecognized. Electrocardiographically, affected patients may demonstrate an array of abnormalities, ranging from isolated conduction delays and first-degree atrioventricular (AV) block to atrial fibrillation, ventricular ectopy, and global repolarization changes suggestive of myocarditis [5]. Myocardial inflammation and ion-channel dysfunction contribute to this variability, and when myocarditis ensues, the risk of malignant arrhythmias increases substantially; reported mortality in leptospiral myocarditis is about 54% [6]. This underscores the importance of early detection of cardiac involvement, particularly in resource-limited settings where clinical deterioration may be rapid.

Despite the high burden of leptospirosis in tropical regions, there exists a notable paucity of systematic studies from India and the broader South Asian region, which together represent a significant proportion of global cases [7]. In India, the southern states like Tamil Nadu and Kerala seem to have the highest burden [7]. There have been ECG studies in the past evaluating the ECG changes in various other cohorts [6], but no robust evidence exists for the Indian population. Given the endemicity, environmental risk factors, and substantial disease burden in this population, understanding the pattern and prognostic significance of electrocardiographic abnormalities is essential. This cross-sectional study aims to address this gap by characterizing the spectrum of ECG changes, such as ST-T changes and bradyarrhythmia, which are observed in patients with laboratory-confirmed leptospirosis and exploring their potential clinical implications.

## Materials and methods

The study received approval from the Institutional Ethics Committee at Madras Medical College, Chennai, India (Approval Number: 009012024). Ethical principles were followed throughout, and written informed consent was obtained from the patient or their legally appointed representative (LAR) in their native language prior to inclusion.

This was a hospital-based clinical observational study conducted over a six-month period at Rajiv Gandhi Government General Hospital, a tertiary care center in Chennai. The study took place from January 2024 to June 2024. All consecutive patients presenting during the study period were screened for eligibility. Patients were included if they had serologically or microscopically confirmed leptospirosis, established through IgM enzyme-linked immunosorbent assay (ELISA), microscopic agglutination test (MAT), or direct microscopic or PCR-based identification, based on the availability of testing kits and standard diagnostic titers. Only patients for whom a standard 12-lead ECG was recorded on admission, as part of routine clinical care, were eligible for analysis. To minimize confounding from prior cardiac disease, individuals with known ischemic heart disease, pre-existing myocarditis or cardiomyopathy, congenital heart defects, documented arrhythmias, accessory pathway disorders such as Wolff-Parkinson-White syndrome, and patients on drugs that can cause ECG changes or electrolyte abnormalities due to non-leptospiral causes were excluded. Patients on maintenance dialysis were also excluded due to inherent ECG and electrolyte variability.

A total of 108 patients fulfilled the inclusion criteria, which is the number of patients admitted to our hospital during the study period. For each participant, demographic variables such as age and sex, clinical severity of leptospirosis, and admission laboratory values were recorded, with particular attention to serum sodium (Na⁺) and potassium (K⁺) levels. All patients underwent a standard 12-lead ECG at the time of admission, and parameters, including rhythm, heart rate, PR interval, QRS duration, QT and QTc intervals, axis, ST-segment deviations, T-wave morphology, conduction blocks, and supraventricular or ventricular arrhythmias, were documented. ECGs were interpreted independently by two trained and blinded physicians, with discrepancies resolved by consensus. Tachycardia was defined as a heart rate of more than 100 bpm; myocarditis-like changes were defined by diffuse and non-specific ST-T changes; the Cornell criteria used the left ventricular hypertrophy (LVH) criteria. QTc interval was calculated using Bazett's formula. Patients were classified as severe or non-severe based on the requirement of admission to the ICU, which was in turn decided by the modified Faine's criteria, symptomatology, and expert clinical judgment.

All collected data were compiled, cleaned, and analyzed using IBM SPSS Statistics for Windows, version 24.0 (IBM Corp., Armonk, NY). Descriptive statistics were used to summarize demographic characteristics, electrolyte values, and ECG findings, with continuous variables expressed as mean ± standard deviation and categorical variables as frequencies and percentages. Differences in ECG abnormalities across clinical severity groups were compared using chi-square tests for categorical variables and independent t-tests or Mann-Whitney U tests for continuous variables, depending on distribution. Spearman rank analyses were performed to evaluate relationships between electrolyte levels and ECG parameters such as conduction intervals and QTc duration. A p-value of <0.05 was considered statistically significant.

## Results

A total of 108 patients with confirmed leptospirosis were included in the study. The mean age of the participants was 34 ± 4.76 years, and the sex distribution was equal, with 54 males and 54 females. Based on clinical criteria, 43 patients (39.8%) required ICU admission due to the severe nature of their symptoms, and the remaining 65 patients (60.2%) were categorized as mild to moderate. There were 14 deaths (13.0%), all occurring in the severe subgroup (Table 1).

**Table 1:** Demographic characteristics of the study population.

Parameter	Value
Age (years), mean ± SD	34 ± 4.76
Sex distribution (M/F)	54/54
Clinical severity	
– No requirement for ICU	65 (60.2%)
– ICU admissions	43 (39.8%)
Mortality	14 (13.0%)
Serum sodium (mmol/L), mean ± SD	132 ± 4.1
Hyponatremia (<135 mmol/L)	72 (66.7%)
Serum potassium (mmol/L), mean ± SD	3.1 ± 0.42
Hypokalemia (<3.5 mmol/L)	81 (75.0%)

Laboratory evaluation on admission revealed a predominant electrolyte disturbance pattern characterized by hyponatremia, with a mean serum sodium level of 132 ± 4.1 mmol/L, and hypokalemia, with a mean potassium level of 3.1 ± 0.42 mmol/L. Hyponatremia was present in 72 patients (66.7%), and hypokalemia in 81 patients (75.0%) (Table 1).

Electrocardiographic abnormalities were seen in 67 patients (62%). Out of which, the most frequent pattern observed was that of ventricular repolarization changes, including nonspecific ST-segment deviations and T-wave inversions, documented in 64 patients (59.3%). A sinus tachycardia with voltage criteria suggestive of LVH (likely reflecting sympathetic overactivity rather than structural disease) was seen in 47 patients (43.5%). Atrial fibrillation was noted in 22 patients (20.4%), while first-degree AV block occurred in 15 patients (13.9%). Higher-grade AV blocks were rare, with second-degree AV block identified in four patients (3.7%). QTc prolongation was infrequent, occurring in only six patients (5.6%) (Table 2).

**Table 2:** ECG changes noted in the study population. LVH: left ventricular hypertrophy; AV: atrioventricular; PVCs: premature ventricular contractions.

ECG finding	Frequency, n (%)
Ventricular repolarization changes	64 (59.3%)
Sinus tachycardia with LVH-like pattern	47 (43.5%)
Atrial fibrillation	22 (20.4%)
First-degree AV block	15 (13.9%)
Second-degree AV block	4 (3.7%)
QTc prolongation	6 (5.6%)
Ventricular ectopy (PVCs)	8 (7.4%)
Normal ECG	12 (11.1%)

Arrhythmias and conduction abnormalities demonstrated clear associations with disease severity. Myocarditis-pattern ECG abnormalities were significantly more common among severe cases compared to non-severe cases (81.4% vs. 44.6%, p < 0.01) (Table 3). Similarly, atrial fibrillation occurred predominantly in the severe group (30.2% vs. 13.8%, p = 0.03). QTc prolongation, although uncommon overall, was observed almost exclusively in severe leptospirosis (9.3% vs. 3.1%), though this trend did not reach statistical significance (Table 4). Hypokalemia showed a moderate correlation with both PR prolongation and the occurrence of supraventricular arrhythmias (p < 0.05), while hyponatremia did not independently predict any specific ECG pattern (Table 5).

**Table 3:** Comparison of ECG abnormalities between the severity groups. Hyphenation indicates that the test was not performed due to low values. AV: atrioventricular.

ECG abnormality	Severe (n = 43)	Non-severe (n = 65)	χ² value	p-value
Myocarditis pattern	35 (81.4%)	29 (44.6%)	13.27	<0.01
Atrial fibrillation	13 (30.2%)	9 (13.8%)	4.72	0.03
QTc prolongation	4 (9.3%)	2 (3.1%)	1.93	0.16
1° AV block	7	8	—	—
2° AV block	3	1	—	—
Ventricular ectopy	4	2	—	—
Ventricular repolarization abnormalities	29	35	—	—

**Table 4:** Correlation between electrolytes and ECG parameters. ρ - Spearman rank correlation coefficient.

ECG parameter	Sodium (Spearman ρ)	p-value	Potassium (Spearman ρ)	p-value
PR interval	-0.10	0.28	-0.32	0.01
QTc duration	0.05	0.61	-0.18	0.07
Supraventricular arrhythmias	0.09	0.32	-0.29	0.02
QRS duration	0.03	0.74	-0.12	0.24

**Table 5:** Electrolyte comparison between the severity groups.

Parameter	Severe (n = 43)	Non-severe (n = 65)	Test	Statistic	p-value
Serum sodium (mmol/L)	131.4 ± 4.2	132.6 ± 4.0	t-test	1.47	0.14
Serum potassium (mmol/L)	3.0 ± 0.40	3.2 ± 0.44	Mann–Whitney	2.21	0.03

Among the 14 patients who died, myocarditis-pattern ECG changes were present in 12 (85.7%), atrial fibrillation in seven (50%), and second-degree AV block in three (21.4%), suggesting a strong association between electrical instability and mortality (Table 6). No malignant ventricular arrhythmias were formally documented, although frequent ventricular ectopy was noted in four of the fatal cases. No multivariate analysis was performed due to the small sample size.

**Table 6:** ECG abnormalities among fatal cases.

ECG abnormality	Fatal cases (n = 14)
Myocarditis-pattern ECG	12 (85.7%)
Atrial fibrillation	7 (50%)
Second-degree atrioventricular block	3 (21.4%)
Ventricular ectopy	4 (28.6%)
Malignant ventricular arrhythmias	0

## Discussion

Leptospirosis remains an important tropical zoonosis with significant systemic involvement, including substantial cardiac manifestations. Although it primarily affects the liver and kidneys, myocardial involvement is increasingly recognized as a major contributor to morbidity and mortality. The spirochetes can directly infiltrate tissues and produce inflammatory cytokine cascades, while electrolyte abnormalities and autonomic dysregulation further predispose patients to conduction disturbances. Because ECG is a rapid, inexpensive, and widely available tool, characterizing its abnormalities in leptospirosis is clinically meaningful, particularly in resource-limited endemic regions.

The study was conducted in the months of January to June, a period of relatively lower prevalence. A sample size of 108 was achieved, owing to the pomposity of the Rajiv Gandhi Government General Hospital (RGGGH), Chennai, which boasts close to 15,000 outpatient visits a day. Another contributing factor could also be the referrals from many peripheral hospitals from all over Tamil Nadu and southern Andhra Pradesh.

In our study, myocarditis-pattern ST-T abnormalities constituted the most frequent ECG finding, followed by sinus tachycardia with LVH-like voltages, atrial fibrillation, and first-degree AV block. QTc prolongation and higher-grade blocks were uncommon. Electrolyte disturbances, specifically hyponatremia and hypokalemia, were highly prevalent and likely contributed to the observed electrical instability. Severe leptospirosis demonstrated a significantly higher burden of arrhythmias, myocarditis-like changes, and conduction defects, and these abnormalities correlated strongly with mortality. The predominance of ST-T changes and supraventricular arrhythmias in fatal cases further highlights the need for vigilant cardiac monitoring in these patients.

Our findings show broad agreement with existing literature. The present study shows a 62% prevalence of ECG changes. A Croatian study reported ST-segment or T-wave abnormalities in 41% of patients, which aligns with the high incidence of myocarditic patterns observed in our cohort [8]. A British comparative study of leptospirosis and malaria showed 56% of subjects exhibited ECG abnormalities [9], underscoring the reproducibility of electrical changes across diverse populations. This mild variation could be due to the fact that our present study included cases from the dry season only, or the fact that the majority of the patient population was from the South Indian race, particularly the Tamil and Telugu populations. The high prevalence of death in the present study (13%) could also stem from many factors, like the fact that RGGGH often gets referrals of serious cases from other hospitals. Another unexplained finding is the high prevalence of atrial fibrillation in our population, which could be due to confounding variables such as unidentified valvular heart lesions, chronic alcoholism, and so on. Further studies could confirm these variations.

Interestingly, a case report described sinus bradycardia as a presenting feature of leptospirosis [10], whereas none of our patients demonstrated bradyarrhythmias, possibly reflecting differing autonomic responses or disease severity profiles. Another study documented ECG abnormalities in 59% of patients but with a lower frequency of myocarditis-like patterns, likely attributable to its smaller sample size [11]. Echocardiographic studies have also shown a spectrum of cardiac involvement: one study reported only 12% of patients with echo findings suggestive of myocarditis [12], while another identified subclinical left ventricular systolic dysfunction, manifesting as abnormal global longitudinal strain despite preserved ejection fraction, as the most common abnormality [13]. Together, these studies reinforce the concept that cardiac involvement in leptospirosis is both common and heterogeneous.

Management of leptospirosis centers on timely antimicrobial therapy and organ support. Intravenous penicillin has long been considered the drug of choice in severe disease, though ceftriaxone and doxycycline are now frequently preferred due to ease of administration and comparable efficacy; ciprofloxacin may be used adjunctively in cases with uveitis [14]. Supportive care remains essential and includes hemodialysis for renal failure, corticosteroids in selected severe systemic inflammatory states like myocarditis, and mechanical ventilation for pulmonary involvement [15]. It is imperative to screen for such illnesses and counsel the patients prior to discharge. Given the cardiac findings outlined in our study, continuous ECG monitoring and prompt correction of electrolyte abnormalities should be incorporated into standard management protocols for moderate and severe cases.

This study has several limitations. It was conducted at a single tertiary center during the dry season, which may limit generalizability to community settings and potentially alter the severity of disease. This could have caused a wrongful depiction of the burden of leptospirosis itself. This could have caused ECGs to be captured only at admission, and the lack of serial follow-up restricts the ability to evaluate the progression or resolution of abnormalities or even underdetection. Echocardiography was not routinely performed, preventing correlation of electrical findings with structural changes. Future research should include multi-center cohorts, potentially conducted in the rainy seasons to incorporate serial ECG and echocardiographic evaluation, and explore the utility of advanced markers such as cardiac MRI or strain imaging to better define the spectrum and prognostic significance of cardiac involvement in leptospirosis.

## Conclusions

Electrocardiographic abnormalities were common in this cohort, with nonspecific ST-T (ventricular repolarization) changes representing the most frequent finding (59.3%). Myocarditis-pattern ECG abnormalities, although not the most prevalent, were strongly associated with severe disease and were present in the majority of fatal cases. The study demonstrated a 13% mortality rate, with deaths showing a high burden of myocarditis-pattern changes (85.7%), atrial fibrillation (50%), and intermittent higher-grade conduction defects, underscoring the prognostic relevance of early ECG assessment. Given the high prevalence of hypokalemia and its correlation with arrhythmias, clinicians should prioritize early identification of myocarditis-like patterns, aggressive correction of electrolyte disturbances, particularly potassium, and continuous ECG monitoring in moderate to severe leptospirosis.

This study represents one of the largest Indian cohorts evaluating ECG abnormalities in leptospirosis and adds important evidence supporting the significance of electrical disturbances as markers of severity and mortality risk. However, the findings must be interpreted in light of key limitations: the study was conducted during the dry, low-prevalence months (January-June), which may not reflect monsoon-season disease patterns, and ECGs were obtained only at admission without serial follow-up or routine echocardiographic correlation. Future research should therefore incorporate serial ECGs, systematic echocardiography, and monsoon season cohorts to better characterize the evolution, mechanisms, and prognostic implications of cardiac involvement in leptospirosis.
